# Viral Detection by Reverse Transcriptase Polymerase Chain Reaction in Upper Respiratory Tract and Metagenomic RNA Sequencing in Lower Respiratory Tract in Critically Ill Children With Suspected Lower Respiratory Tract Infection

**DOI:** 10.1097/PCC.0000000000003336

**Published:** 2023-09-21

**Authors:** Christina M. Osborne, Charles Langelier, Jack Kamm, Kayla Williamson, Lilliam Ambroggio, Ron W. Reeder, Christopher Locandro, J. Kirk Harris, Brandie D. Wagner, Aline B. Maddux, Saharai Caldera, Amy Lyden, Victoria Soesanto, Eric A.F. Simões, Matthew K. Leroue, Todd C. Carpenter, Mark W. Hall, Athena F. Zuppa, Joseph A. Carcillo, Kathleen L. Meert, Murray M. Pollack, Patrick S. McQuillen, Daniel A. Notterman, Joseph DeRisi, Peter M. Mourani

**Affiliations:** 1 Department of Pediatrics, Section of Critical Care Medicine, University of Colorado School of Medicine and Children’s Hospital Colorado, Aurora, CO.; 2 Department of Pediatrics, Section of Infectious Diseases, University of Colorado School of Medicine and Children’s Hospital Colorado, Aurora, CO.; 3 Division of Infectious Diseases, Department of Medicine, University of California San Francisco, San Francisco, CA.; 4 Chan Zuckerberg Biohub, San Francisco, CA.; 5 Department of Biostatistics and Informatics, University of Colorado, Colorado School of Public Health, Aurora, CO.; 6 Department of Epidemiology, University of Colorado School of Medicine, Aurora, CO.; 7 Department of Pediatrics, Section of Emergency Medicine, University of Colorado School of Medicine, Aurora, CO.; 8 Department of Pediatrics, University of Utah, Salt Lake City, UT.; 9 Department of Pediatrics, Section of Pulmonary Medicine, University of Colorado School of Medicine, Aurora, CO.; 10 Division of Critical Care Medicine, Department of Pediatrics, Nationwide Children’s Hospital and The Ohio State University College of Medicine, Columbus, OH.; 11 Anesthesiology and Critical Care, Hospital of the University of Pennsylvania and the Children’s Hospital of Philadelphia, Philadelphia, PA.; 12 Department of Anesthesia and Critical Care Medicine, University of Pittsburgh School of Medicine, Children’s Hospital of Pittsburgh, Pittsburgh, PA.; 13 Department of Pediatrics, Critical Care Medicine, Children’s Hospital of Michigan, Central Michigan University, Detroit, MI.; 14 Department of Pediatrics, Critical Care Medicine, Children’s National Hospital, Washington, DC.; 15 Department of Pediatrics, Benioff Children’s Hospital, University of California, San Francisco, San Francisco, CA.; 16 Department of Molecular Biology, Princeton University, Princeton, NJ.; 17 Department of Pediatrics, Critical Care, University of Arkansas for Medical Sciences and Arkansas Children’s Research Institute, Little Rock, AR.

**Keywords:** diagnostics, metagenomic next-generation sequencing, RNA sequencing, viral lower respiratory tract infection

## Abstract

**OBJECTIVES:**

Viral lower respiratory tract infection (vLRTI) contributes to substantial morbidity and mortality in children. Diagnosis is typically confirmed by reverse transcriptase polymerase chain reaction (RT-PCR) of nasopharyngeal specimens in hospitalized patients; however, it is unknown whether nasopharyngeal detection accurately reflects presence of virus in the lower respiratory tract (LRT). This study evaluates agreement between viral detection from nasopharyngeal specimens by RT-PCR compared with metagenomic next-generation RNA sequencing (RNA-Seq) from tracheal aspirates (TAs).

**DESIGN:**

This is an analysis of of a seven-center prospective cohort study.

**SETTING:**

Seven PICUs within academic children’s hospitals in the United States.

**PATIENTS:**

Critically ill children (from 1 mo to 18 yr) who required mechanical ventilation via endotracheal tube for greater than or equal to 72 hours.

**INTERVENTIONS:**

We evaluated agreement in viral detection between paired upper and LRT samples. Results of clinical nasopharyngeal RT-PCR were compared with TA RNA-Seq. Positive and negative predictive agreement and Cohen’s Kappa were used to assess agreement.

**MEASUREMENTS AND MAIN RESULTS:**

Of 295 subjects with paired testing available, 200 (68%) and 210 (71%) had positive viral testing by RT-PCR from nasopharyngeal and RNA-Seq from TA samples, respectively; 184 (62%) were positive by both nasopharyngeal RT-PCR and TA RNA-Seq for a virus, and 69 (23%) were negative by both methods. Nasopharyngeal RT-PCR detected the most abundant virus identified by RNA-Seq in 92.4% of subjects. Among the most frequent viruses detected, respiratory syncytial virus demonstrated the highest degree of concordance (κ = 0.89; 95% CI, 0.83–0.94), whereas rhinovirus/enterovirus demonstrated lower concordance (κ = 0.55; 95% CI, 0.44–0.66). Nasopharyngeal PCR was more likely to detect multiple viruses than TA RNA-Seq (54 [18.3%] vs 24 [8.1%], *p* ≤ 0.001).

**CONCLUSIONS:**

Viral nucleic acid detection in the upper versus LRT reveals good overall agreement, but concordance depends on the virus. Further studies are indicated to determine the utility of LRT sampling or the use of RNA-Seq to determine LRTI etiology.

**RESEARCH IN CONTEXT**

- Lower respiratory tract infections (LRTIs) are a significant cause of morbidity and mortality, and the majority of these are viral in origin.
- Diagnosis is typically performed with reverse transcriptase polymerase chain reaction (RT-PCR) of upper respiratory samples; however, it is not clear if the detection of a virus in the nasopharynx correlates with virus in the lower respiratory tract at the site of infection.
- This study evaluates the concordance between viral detection in the upper and LRT in children who required mechanical ventilation.

**AT THE BEDSIDE**

- Detection of viral nucleic acid in the upper respiratory tract (URT) demonstrates overall good agreement with the LRT but varies by virus.
- Multiple viruses were detected in the LRT much less frequently than in the URT, and with rare exceptions, only one virus was dominant by quantity.
- Nasopharyngeal samples may be a reasonable surrogate for LRT findings in critically ill pediatric patients with suspected LRTI.

Lower respiratory tract infections (LRTIs) are a common cause of hospital admissions among children and are associated with significant morbidity and mortality. These infections account for 12.8–15.6% of deaths in children less than 5 years of age worldwide (1–3). Viruses account for the majority of LRTIs in children (2–4) and are mostly diagnosed by multiplex reverse transcription polymerase chain reaction (RT-PCR) performed on nasopharyngeal specimens in hospitalized patients (5–7) due to the invasive nature of lower respiratory tract (LRT) sampling. Even for intubated patients, nasopharyngeal specimens are typically utilized for RT-PCR testing as most clinical tests are not approved for use on LRT samples. However, the degree to which RT-PCR results from nasopharyngeal samples reflects presence or absence of virus in the LRT is unclear (8). In particular, the detection of asymptomatic shedding of viruses in the upper respiratory tract (URT) is well-described, potentially resulting in false-positive tests (5–7, 9, 10). Furthermore, it is unclear whether a negative nasopharyngeal RT-PCR reflects the absence of the virus in the LRT or whether a positive test with multiple viruses implies infection by all viruses (11–13).

Understanding the utility of upper airway testing to assess the presence of virus in the lower airways in patients with suspected viral LRTI (vLRTI) has broad implications and relevance to hospital epidemiology and infection control (14–16). If upper airway sampling and microbiologic testing are indeed representative of virus presence in the LRT, clinicians could have more confidence in the results of this testing approach. Furthermore, this comparison could inform the degree to which asymptomatic shedding occurs from the URT in a critically ill population (5–7).

We evaluated the concordance between viral testing of paired upper and LRT samples in a secondary analysis of a multicenter cohort of pediatric patients with respiratory failure requiring mechanical ventilation (MV). We compared the results of clinician-ordered viral RT-PCR testing of nasopharyngeal samples to viral detection by metagenomic next-generation RNA Sequencing (RNA-Seq) performed on paired LRT samples collected via standardized tracheal aspirate (TA) for research purposes. We hypothesized that concordance would vary by virus type and that RNA-Seq may provide data to identify the primary pathogen when multiple viruses are detected.

## MATERIALS AND METHODS

### Study Subjects

This study was a secondary analysis of a prospective multicenter cohort study of mechanically ventilated children admitted to seven PICUs in the National Institute of Child Health and Human Development’s Collaborative Pediatric Critical Care Research Network (CPCCRN) between February 2015 and December 2017 (17). The initial single-site study (site 1) was approved by the Colorado multiple institutional review board ([COMIRB] 14-1530: Microbiome, Virome, and Host Responses Preceding Ventilator-Associated Pneumonia [VAP], originally approved on September 11, 2014), and subsequently, the multiple site study was approved by the CIRB at the University of Utah ([CIRB] CPCCRN 065: Microbiome, Virome and Host Responses Preceding VAP, originally approved on March 4, 2016). Informed consent for all procedures were obtained as a part of the parent study, and procedures are listed therein (17). Procedures were followed in accordance with the ethical standards of the responsible committee on human experimentation (institutional or regional) and with the Helsinki Declaration of 1975. Permission for participation in the study was obtained from the patients’ parents or legal guardians.

The parent study (17) was designed to evaluate the role of the lower airway microbiome and virome in patients at high risk for VAP. Patients ages 31 days to 18 years who were expected to receive MV support via an endotracheal tube (ETT) for more than 72 hours were eligible for inclusion. Exclusion criteria included: children in whom ETT aspirate was not obtained within 24 hours of intubation, those with a tracheostomy tube or plans to place one, conditions in which deep tracheal suctioning was contraindicated, a previous episode of MV during the hospitalization, previous enrollment in the parent study, and limitations of care. Enrolled subjects were eligible for inclusion in this secondary analysis if they had clinician-ordered microbiologic testing using RT-PCR of nasopharyngeal specimens performed within 48 hours of intubation, details of the testing platform were available, a paired TA RNA-Seq result was available, and the subject remained mechanically ventilated for more than 72 hours. This secondary analysis was designed after the completion of the parent study and TA sample analysis via RNA-Seq and thus represents samples with clinical testing available. A subset of samples in the parent study with RNA-Seq results available had RT-PCR testing performed on the TA sample to determine sensitivity of RNA-Seq for the detection of common viruses. Primary admission diagnosis was obtained via chart abstraction from physician documentation. To capture patients who may have had LRTI that was not captured as the primary diagnosis, all patients medical records were reviewed to identify a documented diagnosis of LRTI within 48 hours of admission. All clinical microbiologic results performed within 48 hours of admission were reviewed; although, given the limitations of physician documentation, the precise etiology of the LRTI could not be reliably determined in all cases.

### Sample Collection

Research TA samples were collected within 24 hours of intubation from routine suctioning of the ETT via sterile specimen trap (17). Each site’s personnel were trained in the collection procedure utilizing universal training materials and processes. TA specimens were aliquoted into a tube pre-filled with DNA/RNA Shield (Zymo Research, Irvine, CA) and then frozen at –80°C until analysis.

### RT-PCR Testing

Subjects underwent clinical microbiologic testing of nasopharyngeal samples at the discretion of the treating providers according to the standard of care at each study site. If two respiratory pathogen tests were sent within the inclusion window, the sample collected closer to the time of intubation was utilized. RT-PCR testing was performed by the clinical microbiology lab at each site per their standard operating procedures. A complete list of viral targets by panel and site is listed in **Supplemental Table 1** (http://links.lww.com/PCC/C406). A subset of TA samples from site 1 had sufficient remaining samples after RNA-Seq was performed on which RT-PCR was also performed (Luminex XTAG Respiratory Pathogen Panel; Austin, TX or Biofire FilmArray Pneumonia Panel; Biofire Diagnostics LLC, Salt Lake City, UT). Tests were considered positive if at least one virus was detected. Only qualitative results were available for analysis. Concordance was only evaluated for the viruses that could be detected by the RT-PCR performed. Commercial RT-PCR assays do not differentiate rhinovirus from enterovirus, thus positive results for these viruses are presented as “Rhinovirus/Enterovirus.”

### Identification of Viruses With RNA-Seq

RNA extracted from TA samples underwent library preparation and Illumina paired-end sequencing as previously described (18). Following demultiplexing, raw sequencing reads were host-filtered and quality-filtered and then subjected to viral reference-based alignment using the open-source ID-Seq pipeline (19). This bioinformatics pipeline performs subtractive alignment of the human genome, quality filtering, alignment against the NCBI nucleotide database, and de-novo assembly of reads (19).

Negative control water samples enabled estimation of the number of background reads to each virus. Viruses with sequencing read significantly greater compared with negative controls (adjusted *p* value < 0.05 using a Holm-Bonferroni correction within each sample) were identified by modeling the number of background reads as a negative binomial distribution with mean and dispersion fitted on the negative controls. Viral sequence read numbers were normalized to total sequencing reads per sample including human reads, and reported as reads per million (rpm) reads. An average of 77.8 million reads were obtained per sample (95% CI, 74.7–80.8 million).

### Statistical Analysis

Agreement between viral detection in the paired samples was evaluated using positive and negative percent agreement and Cohen’s Kappa (κ) with 95% CIs for viruses that were detectable with the RT-PCR panel used on that sample. The denominator for each virus was determined by the number of tests with which the virus could possibly be detected. Nasopharyngeal samples were considered the reference standard. McNemar’s chi-square test compared the detection frequency of multiple viruses between samples. A log linear-mixed model was tested the association between the quantity of viruses bymetagenomic next generation RNA sequencing (RNA-Seq) and concordance between nasopharyngeal and TA sample results and included a random subject intercept to account for multiple viruses detected within the same sample.

## RESULTS

### Study Population

Of the 454 patients included in the parent study, 295 (65.0%) had clinical nasopharyngeal RT-PCR testing performed within 48 hours of intubation and a TA RNA-Seq sample with results available (**Supplemental Fig. 1**, http://links.lww.com/PCC/C406). The median time difference between collection of nasopharyngeal and TA samples was 15.6 hours (interquartile range [IQR] 7.5–24.8). The median age of patients was 13 months (IQR 4–49). Most patients were admitted for medical reasons (287, 97.3%), and the majority (230, 78.0%) received a physician diagnosis of LRTI within 48 hours of admission. The cohort had a median duration of MV of 6 days (IQR 5–8), median hospital length of stay of 16 days (IQR 11–25), and in-hospital mortality rate was 4.7% (**Table 1**).

**TABLE 1.** Demographic Characteristics

Characteristic	All (*n* = 295)
Age at Intubation (mo)	13 (4, 49)^a^
Race^b^	
American Indian or Alaska Native	5 (1.7%)
Asian	13 (4.4%)
Black or African American	59 (20%)
Native Hawaiian or other Pacific Islander	3 (1.0%)
White	186 (63.1%)
Unknown or not reported	36 (12.2%)
Ethnicity	
Hispanic or Latino	54 (18.3%)
Not Hispanic or Latino	236 (80.0%)
Unknown, or not reported	3 (1.0%)
History of prematurity (≤ 36 wk)	88 (29.8%)
Primary admission category	
Medical	287 (97.2%)
Surgical	4 (1.4%)
Trauma	4 (1.4%)
Admit primary diagnosis	
Lower respiratory tract infection	196 (66.4%)
Other	66 (22.4%)
Sepsis	29 (9.8%)
Trauma	4 (1.4%)
Diagnosis of lower respiratory tract infection given within 48 hr	230 (78.0%)
Duration of mechanical ventilation (d)	6 (5, 8)^a^
PICU length of stay (d)	10 (7, 15)^a^
Hospital length of stay (d)	16 (11, 25)^a^
In-hospital mortality	14 (4.7%)

a Median (interquartile range).

b More than one choice could be selected.

Data are presented as *n* (%) unless otherwise noted.

### Viral Detection

Of 295 subjects, 65 (22.0%) patients from site 1 had TA samples with sufficient sample remaining on which RT-PCR was run. Using RT-PCR of TA samples as the gold standard, the sensitivity of RNA-Seq to detect on-panel viruses was 89.1%, which we determined was sufficient to allow for comparison of RNA-Seq to RT-PCR (**Supplemental Table 2**, http://links.lww.com/PCC/C406). Of the 65 subject samples, 57 (87.7%) demonstrated concordance between the detection methods. Among discordant viral detections, the direction of the discordance was relatively balanced (five subjects with PCR+/RNA-Seq^–^ compared with three subjects with PCR–/RNA-seq+).

**Figure 1.**
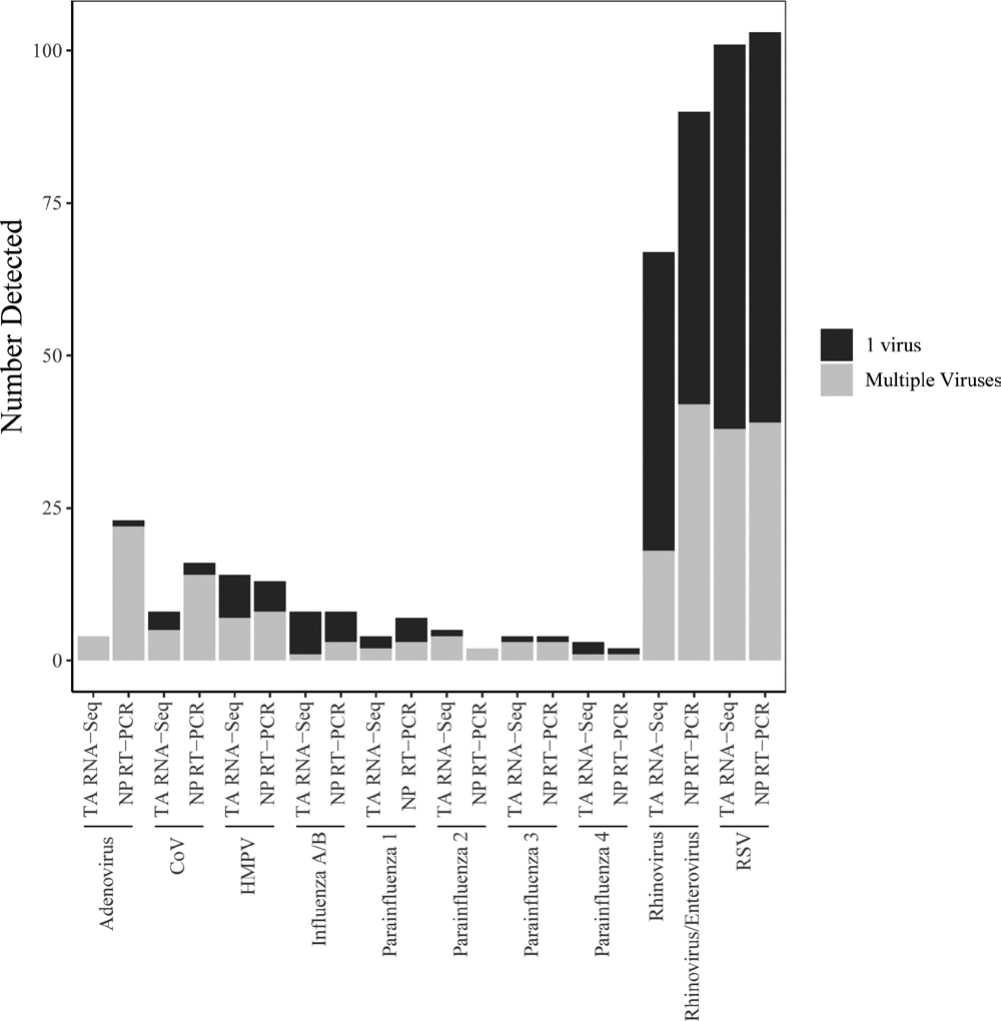
Distribution of viruses detected by commercial multiplex reverse transcriptase polymerase chain reaction (RT-PCR) of nasopharyngeal samples and RNA sequencing (RNA-Seq) of tracheal aspirate (TA) samples (*n* = 295). Within each virus type, samples with a single virus detected are represented in *black*, and samples with multiple viruses detected are designated in *gray*. Samples with multiple viruses detected are included in each individual virus count. RNA-Seq detected viruses not available on the RT-PCR panel in three samples (respiratory syncytial virus [RSV] + Influenza C, RSV + human herpesvirus 6, Parainfluenza 4 + cytomegalovirus). COV = coronavirus (non-SARS CoV-2), HMPV = human metapneumovirus, NP = negative percentage.

Of 295 subjects, at least one virus was detected in 200 (67.8%) nasopharyngeal samples via RT-PCR with 88.0% being diagnosed with LRTI within 48 hours of hospital admission. At least one virus was detected in 210 (71.2%) TA samples via RNA-Seq with 85.7% being diagnosed with LRTI within 48 hours of admission. Respiratory syncytial virus (RSV) was the most frequently detected virus (*n* = 103, 51.5% of nasopharyngeal and *n* = 101, 48.1% of TA samples), followed by rhinovirus/enterovirus (*n* = 90, 45% of nasopharyngeal and *n* = 67, 31.9% of TA samples) (**Fig. 1**). Enterovirus was not detected in any TA sample including serotypes known to cause respiratory disease such as enterovirus D68. Rhinovirus/enterovirus was the most common virus detected in patients without an LRTI diagnosis (66.7% RT-PCR, 53.3% RNA-Seq; **Supplemental Table 4**, http://links.lww.com/PCC/C406). In 18 (6.1%) patients, RNA-Seq detected additional viruses not included in RT-PCR panels including parechovirus (1), parvovirus (2), bocavirus (5), influenza C (3), and human herpes viruses (7). The relative quantity of viruses in the TA samples, measured by RNA-Seq, varied by the patient (**Supplemental Fig. 2**, http://links.lww.com/PCC/C406). Of these 18 patients, 14 (77.8%) had a diagnosis of LRTI, 5 (27.8%) did not have other clinical microbiologic data that would account for their admission diagnosis, and 2 (11.1%) had detection of virus in the lungs that was also detected by PCR of blood (Herpes simplex virus 1, Ebstein Barr virus).

### Testing Agreement

Of the 295 subjects, 184 (62.4%) were positive by both nasopharyngeal RT-PCR and TA RNA-Seq for any virus, with 88.6% being diagnosed with LRTI within 48 hours of admission. For viral targets detectable on RT-PCR, 76 (23.4%) were negative by both methods; 53.7% had a diagnosis of LRTI within 48 hours of admission. With respect to discordant results, 16 (5.4%) of all samples were positive by RT-PCR only; 81.3% had an admission diagnosis of LRTI within 48 hours of admission. Nineteen (6.4%) were positive by RNA-Seq only; 63.2% had a diagnosis of LRTI within 48 hours of admission. Of the 184 samples that were positive by both methods, 171 (92.9%) had at least one detected virus in common and 122 (66.3%) demonstrated complete concordance with all viruses detected. Evaluation of agreement only included viruses tested for by RT-PCR on a given sample. Upper airway RT-PCR detected the most abundant virus detected by RNA-Seq in 92.4% of samples (**Table 2**). Of note, analysis for correlation between patient variables including age, admission category, admission diagnosis, and time between sample collection was performed and only demonstrated statistically significant correlation between younger age and longer time between collection of samples with likelihood of detecting at least one virus the same between samples but not all viruses detected the same (**Supplemental Table 3**, http://links.lww.com/PCC/C406).

**TABLE 2.** Agreement Between Reverse Transcriptase Polymerase Chain Reaction of Nasopharyngeal Samples and RNA Sequencing of Tracheal Aspirate Samples in Samples That Were Positive for Virus That was Detectable by Reverse Transcriptase Polymerase Chain Reaction Testing Performed

Test Results	Number of Paired Samples, *n* (%)
Reverse transcriptase polymerase chain reaction^+^/RNA-Seq^+^ (*n* = 295)	184 (62.3%)
All viruses are the same (*n* = 184)	122 (66.3%)
At least 1 virus is the same (*n* = 184)	171 (92.9%)
Highest quantity of virus (RNA-Seq) present; detected by both methods (*n* = 184)	170 (92.4%)

RNA-Seq = RNA sequencing.

With respect to agreement between paired samples based on type of virus, RSV and human metapneumovirus (HMPV) demonstrated the highest percent positive agreement and highest concordance (Cohen’s kappa (κ) = 0.88; 95% CI, 0.82–0.94 and κ = 0.81; 95% CI, 0.64–0.97, respectively). Influenza demonstrated the next highest level of agreement followed by rhinovirus/enterovirus and coronaviruses. Adenovirus, a DNA virus, was found more frequently in nasopharyngeal samples and demonstrated high negative percent agreement but poor overall agreement (**Table 3**). TA samples with a higher quantity of virus via RNA-Seq were more likely to have concordance with the nasopharyngeal RT-PCR sample, such that concordant samples had 0.91 log_10_(1+rpm) higher quantity of viral load compared with discordant samples (*p* < 0.001; Supplemental Fig. 2, http://links.lww.com/PCC/C406).

**TABLE 3.** Comparison of Reverse Transcriptase Polymerase Chain Reaction and RNA Sequencing Results For Different Viruses

Virus	RT-PCR^+^	RT-PCR^–^	RT-PCR^+^	RT-PCR^–^	Cohen’s Kappa (95% CI)	PPA (95% CI)	NPA (95% CI)
	RNA-Seq^–^	RNA-Seq^+^	RNA-Seq^+^	RNA Seq^–^			
Rhinovirus/Enterovirus	37	14	53	191	0.56 (0.46 to 0.67)	58.2% (47–68%)	92.6% (88–96%)
Respiratory syncytial virus	9	7	94	185	0.88 (0.82 to 0.94)	91.3% (84–96%)	96.4% (93–99%)
Adenovirus	21	2	2	270	0.13 (–0.05 to 0.31)	8.7% (1–28%)	99.2% (97–100%)
Coronavirus	9	1	7	278	0.57 (0.33 to 0.81)	47.1% (23–72%)	99.6% (97–100%)
Human metapneumovirus	2	3	11	279	0.81 (0.064 to 0.97)	84.6% (54–98%)	98.9% (97–100%)
Influenza A/B	2	3	5	285	0.66 (0.38 to 0.94)	62.5% (24–91%)	98.9% (97–100%)

NPA = negative percent agreement, concordant negative/all negative reverse transcriptase polymerase chain reaction (RT-PCR) results, PPA = positive percent agreement, concordant positive/all positive RT-PCR results.

Cohen’s Kappa used to evaluate agreement with 1.00 representing perfect agreement between tests with values ≤ 0 indicating no agreement, 0.01–0.20 as none to slight agreement, 0.21–0.40 as fair agreement, 0.41–0.60 as moderate agreement, 0.61–0.80 as substantial agreement, and 0.81–1.00 as almost perfect agreement.

### Detection of Multiple Viruses

**Figure 2.**
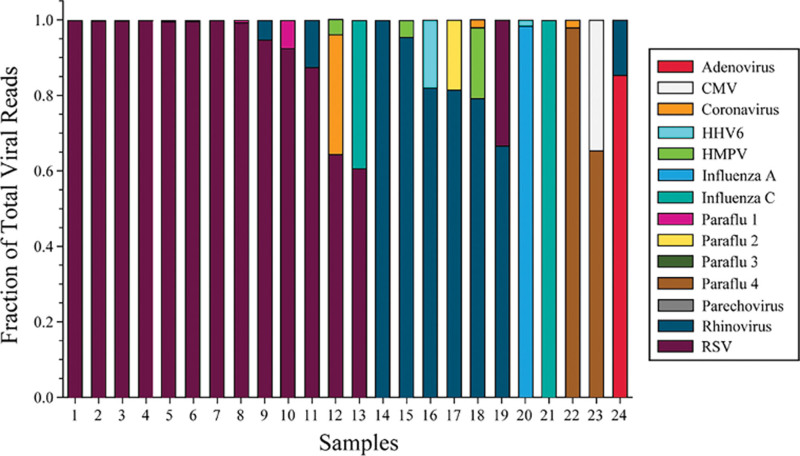
Relative quantity of each virus presents in the 24 RNA sequencing samples that had multiple viruses detected. Each color represents a different virus and each color within a sample bar represents the fraction of total viral reads per million reads (rpm) in each. The total rpm per virus is detailed in Supplemental Table 5 (http://links.lww.com/PCC/C406). CMV = cytomegalovirus, HHV6 = human herpesvirus 6, HMPV = human metapneumovirus, RSV = respiratory syncytial virus.

Multiple viruses were detected in more than twice as many nasopharyngeal samples (*n* = 54 [18.3%]) as TA samples (*n* = 24 [8.1%]; *p* = 0.0004), with more nasopharyngeal samples also having more than two viruses detected (nasopharyngeal: *n* = 13 [4.4%] vs TA: *n* = 2 [0.6%]; *p* = 0.004). In samples with multiple viruses, Rhinovirus/Enterovirus was most commonly identified in nasopharyngeal samples and Rhinovirus in TA samples. In nasopharyngeal samples, RSV and Rhinovirus/Enterovirus were commonly detected together (*n* = 22/54 [40.7%] vs TA samples *n* = 4/24 [16.7%]; *p* = 0.04). All TA samples with multiple viruses detected had a dominant virus (> 50% fractional quantity) with the dominant virus constituting greater than 80% of the reads in 20 (83%) samples (**Fig. 2**). In 13 of 14 samples in which RSV was detected with another virus, RSV was the dominant virus (**Supplemental Table 5**, http://links.lww.com/PCC/C406).

## DISCUSSION

In this large, multicenter study of mechanically ventilated critically ill children with suspected LRTI, we compared the performance characteristics of upper respiratory tract (URT) clinical multiplex viral RT-PCR testing with metagenomic RNA-Seq performed on LRT samples. We found that 86% of samples had concordance between positive and negative results for viruses detected by RT-PCR when including only viruses detectable by the RT-PCR panel for the nasopharyngeal sample. By virus, Cohen’s Kappa values are lower than would typically be seen given the high proportion of negative results for most viruses detected on RT-PCR. Most patients with positive testing by either modality had a clinical diagnosis of LRTI, and our results demonstrate overall high concordance between upper airway RT-PCR and the most abundant virus identified by RNA-Seq in the vast majority (92.9%) of lower airway samples. RNA-Seq demonstrates good ability to detect viruses in the LRT despite the fact that it does not provide amplification of viral RNA. Although the concordance varied depending on the virus, these results suggest that detection of RSV or HMPV by nasopharyngeal RT-PCR is likely a reliable indicator of viral presence in the lower airways as suggested by the Pneumonia Etiology Research for Child Health (PERCH) study (7).

We observed that a greater fraction of paired samples had positive nasopharyngeal RT-PCR and negative TA RNA-Seq than vice versa. A higher percentage of those with positive nasopharyngeal RT-PCR had a clinical diagnosis of LRTI than those positive by TA RNA-Seq (81% vs 63%), which could indicate that clinicians were using these positive clinical test results as a part of their diagnostic criteria for LRTI. Furthermore, detection of multiple viruses occurred more than twice as often in nasopharyngeal samples compared with TA samples, suggesting the possibility that some viruses detected in the URT may represent false positives for LRTI and/or prolonged shedding after a URT infection (6). Furthermore, all patients with multiple viruses detected by TA RNA-Seq exhibited a single-dominant virus present at greater than 50% relative abundance with most having greater than 80% relative abundance (Fig. 2). Although it is difficult to interpret the clinical impact of virus quantity, these data present the possibility that poly-viral LRTI may be less common than is suggested by nasopharyngeal RT-PCR URT testing indicate. We speculate that the lower fraction of polymicrobial viral recovery from TA samples could be due to sampling more closely to the site of active infection, which may increase specificity of results (6, 20, 21). These findings also could suggest that either nasopharyngeal RT-PCR may be more likely to detect incidental upper respiratory viral carriage of a second or third virus unrelated to LRTI (6) or that RNA-Seq is less sensitive with respect to detecting polymicrobial viral infections. Of note, 53.7% of patients with negative testing by both methods had a clinical diagnosis of LRTI, which could be due to isolated bacterial LRTI but may also speak to the challenges associated with the clinical diagnosis of LRTI.

By design, RNA-Seq is an untargeted assay that allows for detection of all viruses present, and 6.1% of our patients had viruses detected that would not have been found with the standard targets available in clinical RT-PCR panels including bocavirus (*n* = 5) and influenza C virus (*n* = 3), which has not been commonly associated with severe disease in humans (22–24). Of these subjects, 27.7% did not have microbiologic results that would explain their illness. Overall, the potential clinical utility of RNA-Seq for diagnosis of LRTI and ability to provide viral quantity should be explored further for potential relevance to severity of illness and outcomes, but this must be weighed against the significant financial cost of this technology.

In contrast with the RNA viruses, the detection of adenovirus demonstrated poor agreement, consistent with the reported limitations of RNA-Seq to detect DNA viruses (25). This does expose a weakness for the potential use of RNA-Seq in patients with LRTI as adenovirus is commonly implicated in childhood LRTIs. Interestingly, detection of influenza was very low by both testing modalities in this cohort of patients across all sites, which will limit ability to extrapolate agreement to a larger population and compare to previous literature (26). Furthermore, rhinovirus was more often detected exclusively in the upper airway (38 of 295, 13%), which may represent exclusively URT infection, asymptomatic infection, or prolonged upper respiratory shedding (27, 28). Given the limitations of this study to differentiate between asymptomatic detection and infection, future studies should evaluate the human host response in the LRT in patients with suspected LRTI to assess whether viruses detected appear to be causing infection as has been done in adult populations (18).

Limitations of our study include an exclusive focus on critically ill patients requiring prolonged MV, which is not representative of the full spectrum of LRTI. Due to limitations of sample availability for the whole cohort, different diagnostic modalities were used for upper versus lower airway sample testing, which may have impacted results. Although we tried to compensate for this by performing RT-PCR on a subset of TA specimens which revealed high concordance for viral detection, future studies should ideally carry out both single-platform RT-PCR and RNA-Seq on both upper and lower airway samples. Given the lack of a true gold standard for diagnosis, the purpose of the study was not to define LRTI but rather to compare testing modalities. Samples were considered paired if they were obtained within 48 hours of intubation, and whereas most were collected within 24 hours, there may be variability in the amount of virus present across the interval time period, which could impact the ability to detect virus and would impact our agreement evaluation. Additionally, institutions differed on the RT-PCR platforms used for clinical testing. Furthermore, given the qualitative nature of our RT-PCR results, it was difficult to determine asymptomatic carriage versus infection for patients with samples only positive by RT-PCR. Although there were specific protocols in place for collection of nasopharyngeal and TA samples, we cannot assure that all samples were obtained correctly, leading to potential heterogeneity of sample quality.

In conclusion, viral detection by upper respiratory RT-PCR demonstrates overall good concordance with LRT RNA-Seq in pediatric patients requiring MV. These data suggest that negative percentage sampling may be a reasonable surrogate for LRT viral testing. In cases with multiple viruses detected, lower airway RNA-Seq suggests there is often one dominant virus.

## ACKNOWLEDGMENTS

The study investigators thank all subjects and their families for participating in this project. We also acknowledge the contributions of Tammara L. Jenkins, MSN, RN, and Robert F. Tamburro, MD, from Eunice Kennedy Shriver National Institute of Child Health and Human Development, Bethesda, MD. Following is a summary of Performance Sites, Principal Investigators (PI), Co-investigators (CI), Research Coordinators (RC), and Allied Research Personnel. Children’s Hospital of Colorado, Aurora, CO: Peter Mourani (PI); Marci Sontag (PI); Todd Carpenter (CI); Yamila Sierra (RC); Katheryn Malone (RC), Diane Ladell (RC); Kimberly Ralston (RC); Kevin Van (RC). Children’s Hospital of Michigan, Detroit, MI: Kathleen L. Meert (PI); Sabrina Heidemann (CI); Ann Pawluszka (RC); Melanie Lulic (RC). Children’s Hospital of Philadelphia, Philadelphia, PA: Robert A Berg (PI); Athena Zuppa (CI); Carolann Twelves (RC); Mary Ann DiLiberto (RC). Children’s National Medical Center, Washington, DC: Murray Pollack (PI); David Wessel (PI); Randall Burd (CI); Elyse Tomanio (RC); Diane Hession (RC); Ashley Wolfe (RC). Nationwide Children’s Hospital, Columbus, OH: Mark Hall (PI); Andrew Yates (CI); Lisa Steele (RC); Maggie Flowers (RC); Josey Hensley (RC). Mattel Children’s Hospital, University of California Los Angeles, Los Angeles, CA: Anil Sapru (PI); Rick Harrison (CI), Neda Ashtari (RC); Anna Ratiu (RC). Children’s Hospital of Pittsburgh, University of Pittsburgh Medical Center, Pittsburgh, PA: Joe Carcillo (PI); Ericka Fink (CI); Leighann Koch (RC); Alan Abraham (RC). Benioff Children’s Hospital, University of California, San Francisco, San Francisco, CA: Patrick McQuillen (PI); Anne McKenzie (RC); Yensy Zetino (RC). University of Utah; Data Coordinating Center, Salt Lake City, Utah: Mike Dean (PI); Richard Holubkov (PI), Juhee Peterson, Melissa Bolton, Whit Coleman, and Stephanie Dorton.

## Supplementary Material
