# Supplementary material for: Viral Detection by Reverse Transcriptase Polymerase Chain Reaction in Upper Respiratory Tract and Metagenomic RNA Sequencing in Lower Respiratory Tract in Critically Ill Children With Suspected Lower Respiratory Tract Infection
Source: Pediatr Crit Care Med. 2023 Sep 21;25(1):e1–e11. doi: 10.1097/PCC.0000000000003336 (PMC10756702; doi:10.1097/PCC.0000000000003336)
Supplement: Supplementary file 1 [file pcc-25-e1-s001.docx]

**Supplemental Table 1: Clinical RT-PCR testing available at each site in the multi-site CPCCRN study network.** Sites 2 and 5 had option for clinicians to test for only influenza and RSV rather than a multiplex RT-PCR with larger number of targets. RPP = respiratory pathogen panel.

| **Site** | **RPP used** | **RSV/ Influenza PCR** | **Adenovirus** | **RSV** | **Influenza** | **Para-**  **influenza 1** | **Para-**  **influenza 2** | **Para- influenza 3** | **Para-**  **influenza 4** | **Human meta- pneumovirus** | **Rhinovirus/ Enterovirus** | **Coronavirus** |
| --- | --- | --- | --- | --- | --- | --- | --- | --- | --- | --- | --- | --- |
| **1** | **Biofire** | no | yes | yes | yes | yes | yes | yes | yes | yes | yes | yes |
| **2** | **In-house** | yes  (in-house) | yes | yes | yes | yes | yes | yes | yes | yes | yes | no |
| **3** | **Biofire** | no | yes | yes | yes | yes | yes | yes | yes | yes | yes | yes |
| **4** | **Luminex XTAG** | no | yes | yes | yes | yes | yes | yes | no | yes | yes | no |
| **5** | **GenMark XT8** | yes (Cepheid) | yes | yes | yes | yes | yes | yes | no | yes | yes | no |
| **6** | **Biofire RP** | no | yes | yes | yes | yes | yes | yes | yes | yes | yes | yes |
| **7** | **GenMark XT8** | no | yes | yes | yes | yes | yes | yes | no | yes | yes | no |

**Supplemental Table 2: Comparison of lower respiratory tract RT-PCR to RNA-Seq in subset of patients** (n = 65)

| **Virus** | **PCR+** | **RNA-Seq+** | **PCR+/**  **RNA-Seq+** | **PCR+/**  **RNA-Seq-** | **PCR-/ RNA-Seq+** | **PCR-/ RNA-Seq-** | **Sensitivity**  **(95% CI)** | **Specificity**  **(95% CI)** |
| --- | --- | --- | --- | --- | --- | --- | --- | --- |
| **Adenovirus** | 1 | 1 | 0 | 1 | 1 | 63 | 0.0%  (0, 97.5) | 98.4%  (91.6, 100.0) |
| **Coronavirus** | 4 | 4 | 3 | 1 | 1 | 60 | 75.0%  (19.4, 99.4) | 98.4%  (91.2, 100.0) |
| **HMPV** | 5 | 4 | 4 | 1 | 0 | 60 | 80.0% (28.4,99.5) | 100.0%  (94, 100.0) |
| **Rhinovirus/**  **Enterovirus** | 15 | 15 | 12 | 3 | 3 | 47 | 80.0%  (51.9, 95.7) | 94.0%  (83.5, 98.8) |
| **Influenza** | 2 | 0 | 0 | 2 | 0 | 63 | 0.0%  (0, 84.2) | 100.0%  (94.3, 100.0) |
| **Parainfluenza** | 8 | 7 | 6 | 2 | 1 | 56 | 75.0%  (34.9, 96.8) | 98.3%  (90.6, 100.0) |
| **RSV** | 19 | 17 | 16 | 3 | 1 | 45 | 84.21% (60.4, 96.6) | 97.83%  (88.5, 99.9) |
| **Total patients** | 46 | 44 | 41 | 5 | 3 | 16 | 89.13% (76.4, 96.4) | 84.21%  (60.4, 96.6) |

**Supplemental Table 3:** Evaluation of variables that may impact concordance or discordance between samples including time difference between collection of TA samples for RNA-Seq and NP samples for RT-PCR, age, admission category, and admission diagnosis. ^1^At least one virus detected between the two samples were the same; 171 positive by both and 69 negative by both methods

^2^ No viruses detected between the two samples were the same ^3^All viruses detected between the two samples were the same; 122 positive by both and 69 negative by both methods

|  | **At least 1 virus the same** | | | **All viruses the same** | | |
| --- | --- | --- | --- | --- | --- | --- |
|  | **Concordant^1^**  **(n = 240)** | **Discordant^2^**  **(n = 55)** | **p-value** | **Concordant^3^**  **(n = 191)** | **Discordant^2^**  **(n = 88)** | **p-value** |
| Time between collection of TA and NP samples in hours | 14.8 (7.0, 24.8) | 10.9 (0, 17.6) | 0.01 | 13.9 (5.6, 23.7) | 14.0 (4.2, 25.1) | 0.99 |
| Age at Intubation (months) | 10.8 (3.2, 47.3) | 17.0 (6.5, 52.9) | 0.08 | 13.6 (3.4, 56.1) | 10.6 (4.1, 40.4) | 0.63 |
| Primary Admission Category | | | 0.81 |  | | 0.29 |
| Medical | 233 (97.1%) | 54 (98.2%) |  | 184 (96.3%) | 88 (100%) |  |
| Surgical | 4 (1.7%) | 0 |  | 4 (2.1%) | 0 |  |
| Trauma | 3 (1.3%) | 1 (1.8%) |  | 3 (1.6%) | 0 |  |
| Admit Primary Diagnosis | | | 0.47 |  | | 0.67 |
| Lower respiratory tract infection | 163 (67.9%) | 33 (60.0%) |  | 124 (64.9%) | 61 (69.3%) |  |
| Other | 50 (20.8%) | 16 (29.1%) |  | 42 (22.0%) | 20 (22.7%) |  |
| Sepsis | 24 (82.8%) | 5 (9.1%) |  | 22 (11.5%) | 7 (8.0%) |  |
| Trauma | 3 (1.3%) | 1 (1.8%) |  | 3 (1.6%) | 0 |  |

**Supplemental Table 4: Virus detected in patients with a non-LRTI primary admitting diagnosis, comparing those with no LRTI diagnosed within 48 hours to patients with a LRTI diagnosed within 48 hours.** n represents number of patients; patients could test positive for more than 1 virus.

|  | **No LRTI diagnosed within 48 hours (n = 65)** | | **LRTI diagnosed within**  **48 hours (n = 36)** | |
| --- | --- | --- | --- | --- |
| **Viruses** | **RT-PCR+ (n = 24)** | **RNA-Seq+ (n = 30)** | **RT-PCR+ (n = 17)** | **RNA-Seq+ (n = 19)** |
| Multiple viruses | 7 | 0 | 7 | 2 |
| Adenovirus | 3 | 0 | 6 | 2 |
| Coronavirus | 2 | 0 | 0 | 1 |
| Human metapneumovirus | 1 | 1 | 0 | 0 |
| Influenza A/B | 0 | 1 | 3 | 1 |
| Parainfluenza 1 | 2 | 1 | 0 | 0 |
| Parainfluenza 2 | 1 | 1 | 0 | 0 |
| Parainfluenza 3 | 0 | 1 | 1 | 1 |
| Parainfluenza 4 | 0 | 1 | 0 | 0 |
| Rhinovirus/Enterovirus | 16 | 16 | 9 | 8 |
| RSV | 6 | 8 | 4 | 6 |
| Human herpesvirus | N/A | 2 | N/A | 0 |
| Influenza C | N/A | 1 | N/A | 0 |

**Supplemental Table 5: Total viral abundance from RNA-seq in reads per million (rpm) for patients with multiple viruses detected in the lower respiratory tract.** RSV= respiratory syncytial virus; HMPV = human metapneumovirus; HHV6 = human herpes virus 6; CMV = cytomegalovirus; all coronaviruses are non-SARS-CoV-1 or 2 and non-MERS-CoV viruses.

| **Virus 1** | **Virus 1 abundance (rpm)** | **Virus_2** | **Virus 2 abundance (rpm)** | **Virus_3** | **Virus 3 abundance (rpm)** |
| --- | --- | --- | --- | --- | --- |
| RSV | 48782.57 | Parainfluenza 3 | 2.14 |  |  |
| RSV | 49.01 | Rhinovirus | 0.07 |  |  |
| RSV | 2.13 | Rhinovirus | 0.30 |  |  |
| RSV | 69.43 | Coronavirus | 0.09 |  |  |
| RSV | 801.08 | Parainfluenza 2 | 0.10 |  |  |
| RSV | 572.85 | Rhinovirus | 31.16 |  |  |
| RSV | 26.44 | Influenza C | 17.08 |  |  |
| RSV | 659.37 | Coronavirus | 324.67 | HMPV | 39.61 |
| RSV | 135.70 | Parainfluenza 3 | 0.49 |  |  |
| RSV | 95.60 | Adenovirus | 0.23 |  |  |
| RSV | 77.11 | Parainfluenza 1 | 0.48 |  |  |
| RSV | 2523.20 | HHV6 | 0.13 |  |  |
| RSV | 583.68 | Parainfluenza 1 | 46.71 |  |  |
| Rhinovirus | 0.11 | RSV | 0.05 |  |  |
| Rhinovirus | 9287.64 | Parechovirus | 0.65 |  |  |
| Parainfluenza 4 | 85.83 | Coronavirus | 1.78 |  |  |
| Parainfluenza 4 | 0.71 | CMV | 0.38 |  |  |
| Parainfluenza 2 | 46.32 | Rhinovirus | 204.16 |  |  |
| Influenza C | 331.82 | Parainfluenza 2 | 0.07 |  |  |
| Influenza A | 15.68 | HHV6 | 0.25 |  |  |
| HMPV | 9.35 | Rhinovirus | 39.43 | Coronavirus | 1.00 |
| HHV6 | 9.40 | Rhinovirus | 42.80 |  |  |
| Adenovirus | 7.58 | Rhinovirus | 1.30 |  |  |
| Adenovirus | 476.09 | HMPV | 22.20 |  |  |

**Supplemental Figure 1: Study schematic.** Reasons for excluded subjects are not mutually exclusive.

**
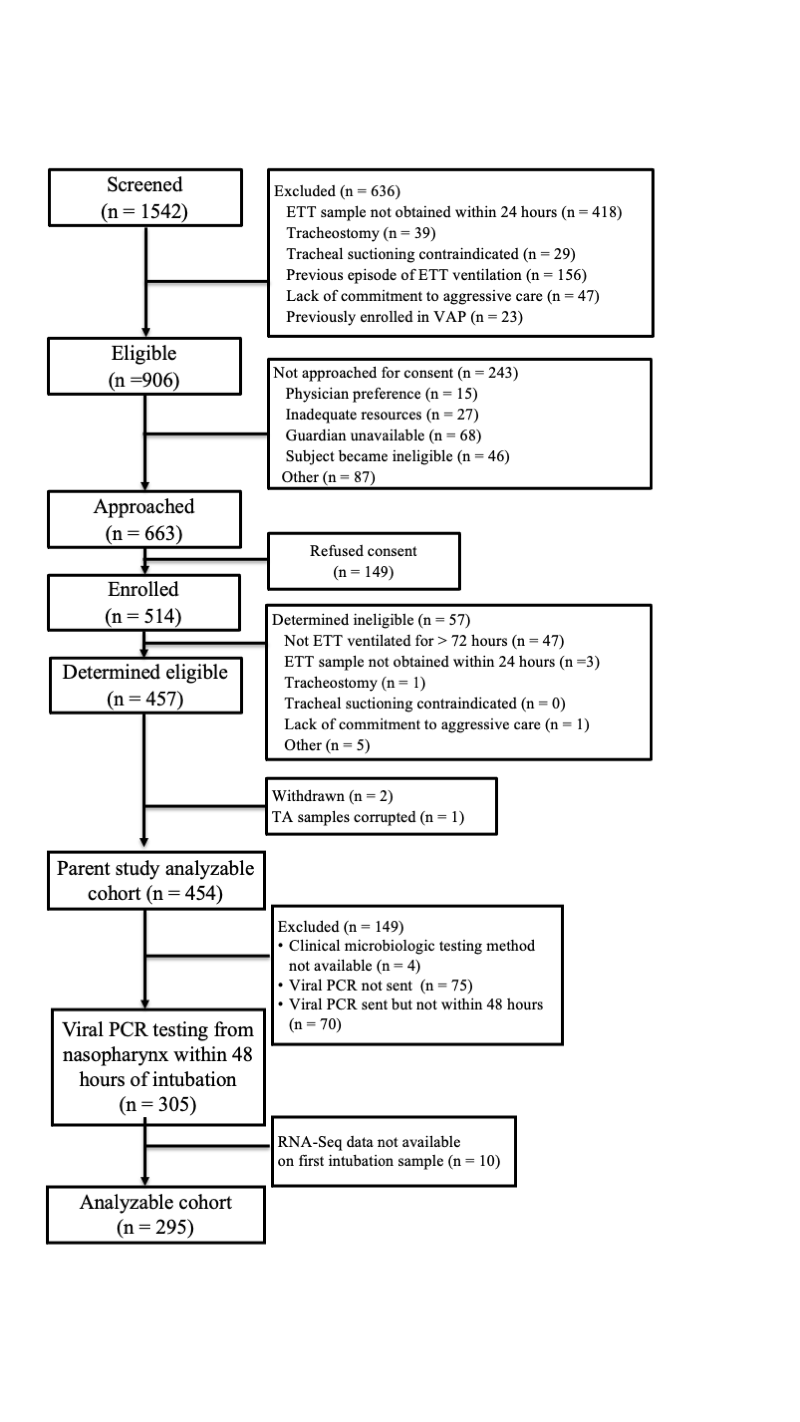
**

**Supplemental Figure 2: Comparison of virus quantity from RNA-seq in reads per million reads (rpm) for concordant samples versus discordant samples.** TA samples with higher viral abundance were more likely to also have the virus be detected in NP samples. There is a statistically significant 0.91 increase in log10 (1+rpm) for concordant samples versus non concordant samples after accounting for repeated viruses detected in a sample (p<0.001). The lower and upper hinges correspond to the first and third quartiles (the 25th and 75th percentiles). The upper whisker extends from the hinge to the largest value no further than 1.5 times the interquartile range (IQR) from the hinge. The lower whisker extends from the hinge to the smallest value at most 1.5 times the IQR of the hinge. Data beyond the end of the whiskers are called "outlying" points and are plotted individually.

**
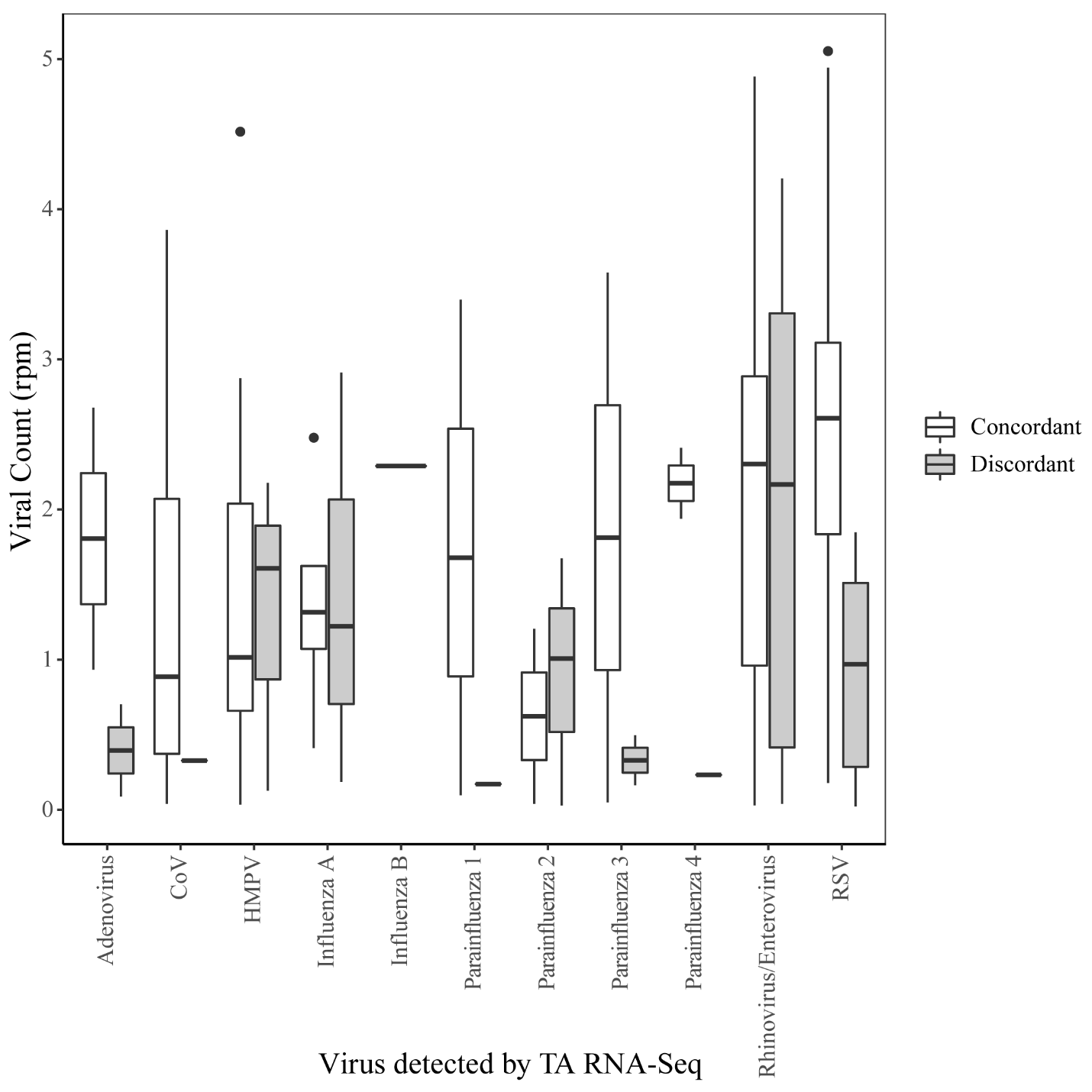
**
